# Study on antibacterial activity of silver nanoparticles synthesized by gamma irradiation method using different stabilizers

**DOI:** 10.1186/1556-276X-9-162

**Published:** 2014-04-04

**Authors:** Dang Van Phu, Le Anh Quoc, Nguyen Ngoc Duy, Nguyen Thi Kim Lan, Bui Duy Du, Le Quang Luan, Nguyen Quoc Hien

**Affiliations:** 1 Research and Development Center for Radiation Technology, Vietnam Atomic Energy Institute, 202A Street 11, Linh Xuan Ward, Thu Duc District, Ho Chi Minh City, Vietnam; 2 Institute of Applied Material Science, Vietnam Academy of Science and Technology, 1 Mac Dinh Chi Street, 1 District, Ho Chi Minh City, Vietnam; 3 Center for Nuclear Technique, Vietnam Atomic Energy Institute, 217 Nguyen Trai Street, 1 District, Ho Chi Minh City, Vietnam

**Keywords:** Silver nanoparticles, Gamma irradiation, Antibacterial, *E. coli*, Handwash

## Abstract

Colloidal solutions of silver nanoparticles (AgNPs) were synthesized by gamma Co-60 irradiation using different stabilizers, namely polyvinyl pyrrolidone (PVP), polyvinyl alcohol (PVA), alginate, and sericin. The particle size measured from TEM images was 4.3, 6.1, 7.6, and 10.2 nm for AgNPs/PVP, AgNPs/PVA, AgNPs/alginate, and AgNPs/sericin, respectively. The influence of different stabilizers on the antibacterial activity of AgNPs was investigated. Results showed that AgNPs/alginate exhibited the highest antibacterial activity against *Escherichia coli* (*E. coli*) among the as-synthesized AgNPs. Handwash solution has been prepared using Na lauryl sulfate as surfactant, hydroxyethyl cellulose as binder, and 15 mg/L of AgNPs/alginate as antimicrobial agent. The obtained results on the antibacterial test of handwash for the dilution to 3 mg AgNPs/L showed that the antibacterial efficiency against *E. coli* was of 74.6%, 89.8%, and 99.0% for the contacted time of 1, 3, and 5 min, respectively. Thus, due to the biocompatibility of alginate extracted from seaweed and highly antimicrobial activity of AgNPs synthesized by gamma Co-60 irradiation, AgNPs/alginate is promising to use as an antimicrobial agent in biomedicine, cosmetic, and in other fields.

## Background

The common stabilizers used in bottom-up approach of metallic nanoparticle synthesis (Au, Pt, Ag, etc.) are polymers and surfactants such as sodium dodecyl sulfate (SDS) and Tween 80 [1]. Furthermore, citrate which is a typical electro-static stabilizer has been popularly used [2]. Followed by citrate, the synthetic polymers have also been widely used as stabilizers, typically polyvinyl alcohol (PVA) [3-5] and polyvinyl pyrrolidone (PVP) [5-9]. On the other hand, among the natural polysaccharide stabilizers, chitosan [10-14] and alginate [15,16] are commonly used. In addition, the derivatives of cellulose such as carboxylmethyl cellulose [1,17], hydroxypropyl cellulose [18], and gelatin [7,19] were also used as stabilizers for the synthesis of metallic nanoparticles.

The study results of Kvítek et al. showed that the antibacterial activity of silver nanoparticles (AgNPs) was significantly enhanced by using the suitable stabilizers such as the following surfactants: SDS, Tween 80, and PVP K90 [1]. Moreover, the study results of El Badawy et al. also showed that AgNPs/PVP had the higher antibacterial activity than AgNPs/citrate and especially AgNPs capped by branched polyethyleneimine (BPEI) had better antibacterial activity than the other ones [20]. They also claimed that the mechanism of AgNP toxicity may involve a combination of both physical and chemical interactions. There was a direct correlation between the toxicity of AgNPs and their surface charge. The more negative the zeta value, the less toxic are the AgNPs to *bacillus* species. The zeta potential of AgNPs/citrate was −38 mV, whereas the zeta potential of AgNPs/PVP and AgNPs/BPEI were −10 and +40 mV, respectively [20]. Therefore, the various stabilizers for AgNPs affect not only on the stability but also on the antibacterial activity of AgNP colloid [1,14,20,21].

In this study, we prepared four colloidal AgNP solutions at a concentration of 1-mM Ag in different stabilizers, namely PVP, PVA, alginate, and sericin with the same concentration of 0.5% (*w*/*v*). Subsequently, the antibacterial activity of these colloidal AgNP solutions was investigated. To further demonstrate the effect of AgNPs on antibacterial activity and apply the development in practice, the AgNPs were added into a handwash solution, and the antibacterial activity was also tested.

## Methods

### Material

Pure-grade AgNO_3_ was purchased from Shanghai Chemical Reagent Co., Shanghai, China The pharmaceutical grade PVP K90 was a product from Merck, Darmstadt, Germany. PVA 217 was a product of Kuraray, Tokyo, Japan. Alginate was a product of Hayashi Pure Chemical Industries, Osaka, Japan, and sericin was purchased from Sigma, St. Louis, MO, USA. Distilled water was used throughout the preparation of colloidal AgNP solutions. The strain of *Escherichia coli* ATCC 6538 was provided by the University of Medical Pharmacy, Ho Chi Minh City. The Luria-Bertani (LB) medium purchased form Himedia, Mumbai, India contains 10 g triptone, 5 g yeast extract, 10 g sodium chloride, and 1 L distilled water.

### Synthesis of AgNPs

Four colloidal solution samples of 1-mM AgNPs stabilized in 0.5% (*w*/*v*) stabilizers of PVP, PVA, alginate, and sericin were prepared by gamma Co-60 irradiation method as described in our previous papers [9,13]. Briefly, the stabilizers were dissolved in water to reach a concentration of 0.5%. AgNO_3_ was then dissolved in the above prepared solution to obtain a final concentration of 1-mM Ag^+^. The mixture was poured into glass bottles with plastic caps. The irradiation of these solutions at dose of 6 kGy for the synthesis of AgNPs was carried out on a Co-60 irradiator with a dose rate of approximately 1.2 kGy/h at VINAGAMMA Center, Ho Chi Minh City. Absorption spectra of the irradiated AgNP solutions with dilution by water to 0.1-mM AgNPs were taken on an UV-vis spectrophotometer, Jasco V-630 (Easton, MD, USA). The AgNP sizes were measured using a transmission electron microscope (TEM; JEM 1010, JEOL, Tokyo, Japan).

### Antibacterial activity of AgNPs

A 99-mL sterilized LB medium and AgNPs in different stabilizers as prepared for the final concentration of 1-mg/L AgNPs were added into conical flasks (250 mL), and the control sample just contained a 99-mL LB medium. A 1-ml *E. coli* suspension (approximately 10^7^ CFU/mL) was added to each flask. The cultures were shaken at 150 rpm, and the bacterial growth curves were determined by measuring optical density (OD) at 600 nm on a UV-vis Jasco V-630 with 30-min interval [11,22,23].

### Bactericidal activity of handwash containing AgNPs

A handwash solution was prepared using Na lauryl sulfate (Na-LS) as surfactant, hydroxyethyl cellulose (HEC) as binder, and 15 mg/L of AgNPs/alginate as antimicrobial agent. The bactericidal activity assay of the handwash against *E. coli* was carried out by culture medium toxicity method [11,13] as follows: the handwash samples (with and without AgNPs) were put into 99-mL LB medium for the final concentration of 3-mg/L AgNPs, whereas the control sample just contains 99-mL LB. Subsequently, 1-mL *E. coli* suspension of 10^7^ CFU/mL was injected to each sample. The samples were shaken at 150 rpm at room temperature for 1, 3, and 5 min. After that, the number of bacteria in each mixture was quantified by spread plate technique on LB agar plates.

## Results and discussion

**Figure 1:**
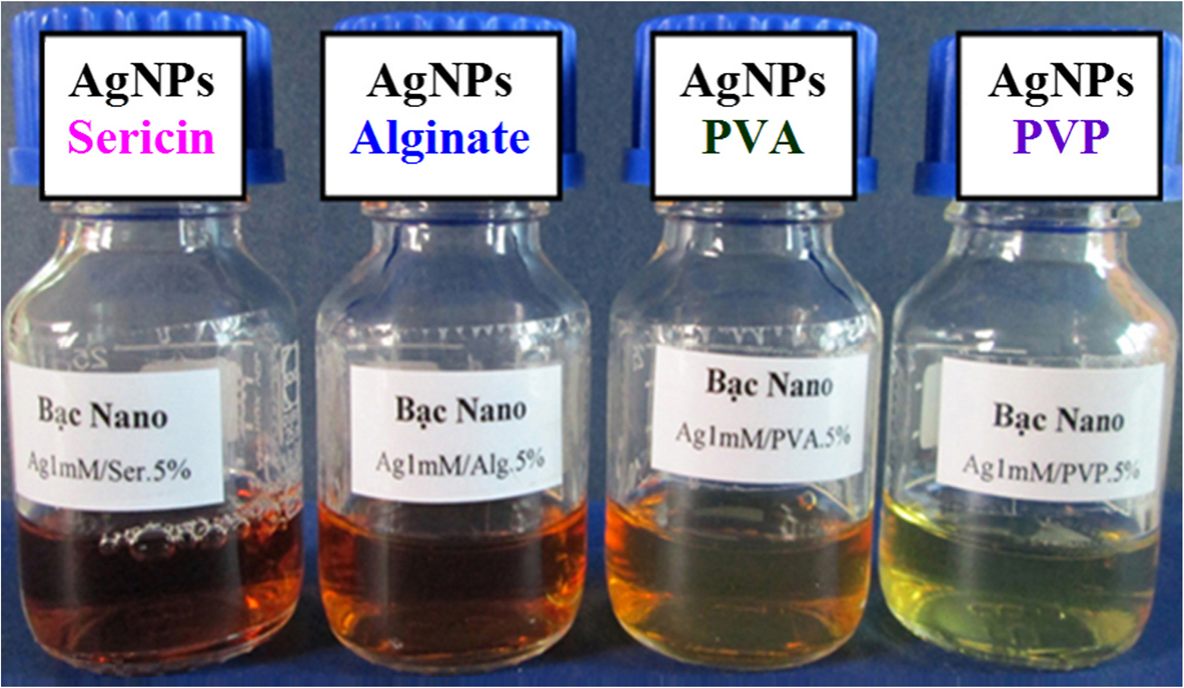
Photograph of 1-mM AgNPs in different stabilizer solutions.

**Figure 2:**
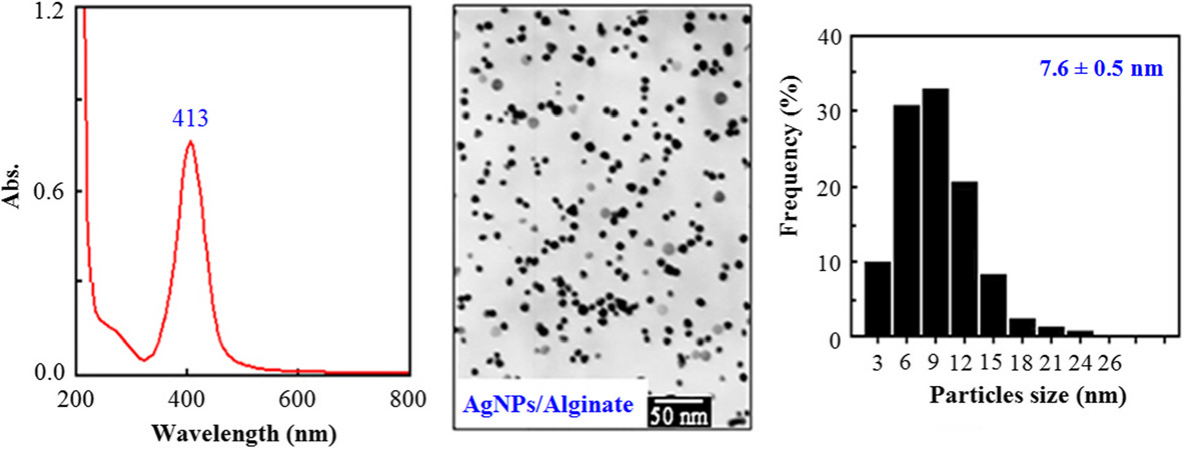
A typical UV-vis spectrum, TEM image, and size distribution of AgNPs/alginate.

The successful synthesis of AgNPs stabilized in different polymer solutions was first revealed by the specific colors that the colloidal AgNP solution displays (Figure 1). A UV-vis spectrum with a maximum wavelength (*λ*_max_) of 413 nm, TEM image with quasi-spherical particles, and narrow size distribution of AgNPs stabilized by alginate were typically described in Figure 2. It is clear that the resulting colloidal solutions exhibited the characteristic surface plasmon resonance (SPR) band of AgNPs with *λ*_max_ at 410 to 420 nm (see Table 1) [4,11].

**Table 1:** **The λ**_**max**_**, OD, and average size (*d *) of the colloidal AgNP solution in different stabilizers**

**Stabilizers**	**λ**_**max **_**(nm)**	**OD**	***d *** **(nm)**
PVA	411	0.80	6.1 ± 0.2
PVP	407	0.65	4.3 ± 0.4
Sericin	418	0.25	10.2 ± 1.1
Alginate	413	0.76	7.6 ± 0.5

The results in Table 1 also indicated that the AgNP average diameters were 6.1, 4.3, 10.2, and 7.6 nm for PVA, PVP, sericin, and alginate stabilizer, respectively. It is obvious that the stabilizers affected the size of AgNPs synthesized by the gamma Co-60 irradiation method. In addition, the stabilizers were also found to influence the stability and antibacterial activity of the AgNPs [1,21,24]. According to Zhang et al., the stability of the colloidal AgNP solutions with different stabilizers was in the following sequence: AgNPs/PVP > AgNPs/casein > AgNPs/dextrin [24]. Furthermore, the results of Liu et al. [15] and Lan et al. [16] also confirmed the good stability of AgNPs synthesized by gamma Co-60 irradiation method using alginate as the stabilizer.

The gamma Co-60 irradiation method is fairly suitable to create the smaller AgNPs compared to chemical reduction method [8]. It is generally admitted that the smaller the AgNPs, the stronger the antibacterial effect. AgNPs have been currently applied as disinfecting agents in general practice due to their antibacterial effects (http://www.nanotechproject.org/inventories/consumer/analysis_draft/). Therefore, antibacterial activity of the resulted AgNP solutions, namely AgNPs/PVA, AgNPs/PVP, AgNPs/sericin, and AgNPs/alginate was tested.

**Figure 3:**
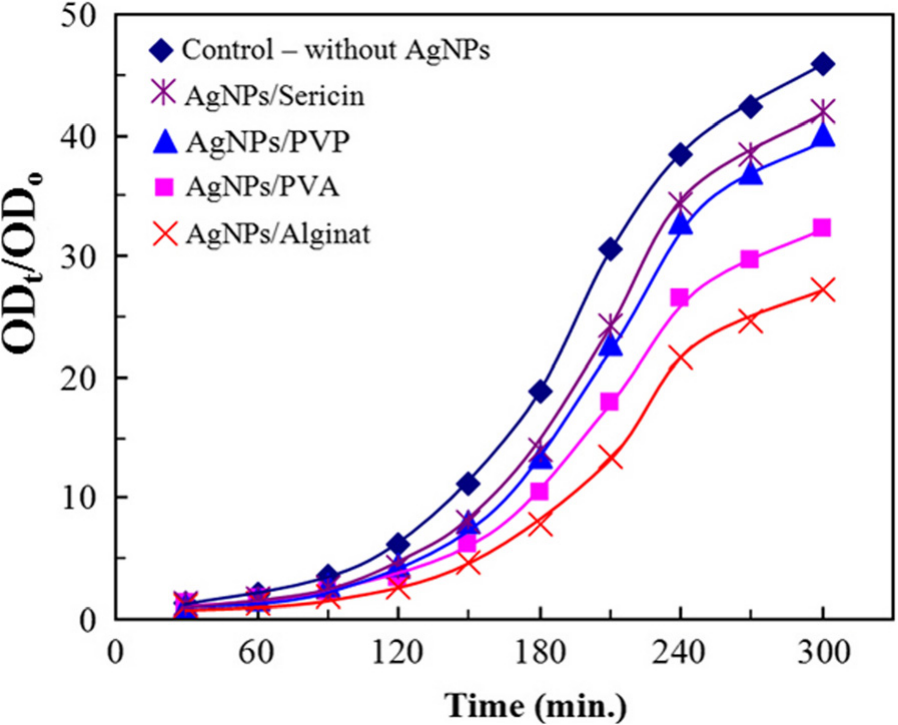
**The growth curves of *E. coli *** **exposed to the colloidal AgNPs in different stabilizers.**

Figure 3 displayed the dynamics of bacterial growth in liquid LB medium supplemented with 10^7^*E. coli* cells/100 mL and 1-mg/L AgNPs in different stabilizers. OD_*o*_ and OD_*t*_ (Figure 3) are the optical density values of the studied sample solutions at the beginning and at the different contacting time, respectively. In all AgNP-treated samples, the AgNPs caused a growth delay of *E. coli* compared with the control sample, and the growth delay effect was different in the following sequence: AgNPs/alginate (7.6 nm) > AgNPs/PVA (6.1 nm) > AgNPs/PVP (4.3 nm) > AgNPs/sericin (10.2 nm). The obtained results also proved that the antibacterial effect of AgNPs depends not only on the size but also on the stabilizer used.

In addition, Sondi and Salopek-Sondi [25] and Tiwari et al. [22] reported that the concentration of AgNPs is mainly responsible for the antibacterial effect along with treatment time. Moreover, the results of El Badawy et al. have also confirmed that the stabilizers of the AgNPs were one of the most important determinants of the antibacterial activity of AgNPs [20]. For that reason, upon each application purpose, the appropriate stabilizer should be chosen for capping AgNPs, especially for applying AgNPs as antibacterial agents. Therefore, in this study, an antibacterial handwash solution was prepared using Na-LS as surfactant, HEC as binder, and 15 mg/L of AgNPs/alginate as antimicrobial agent.

**Figure 4:**
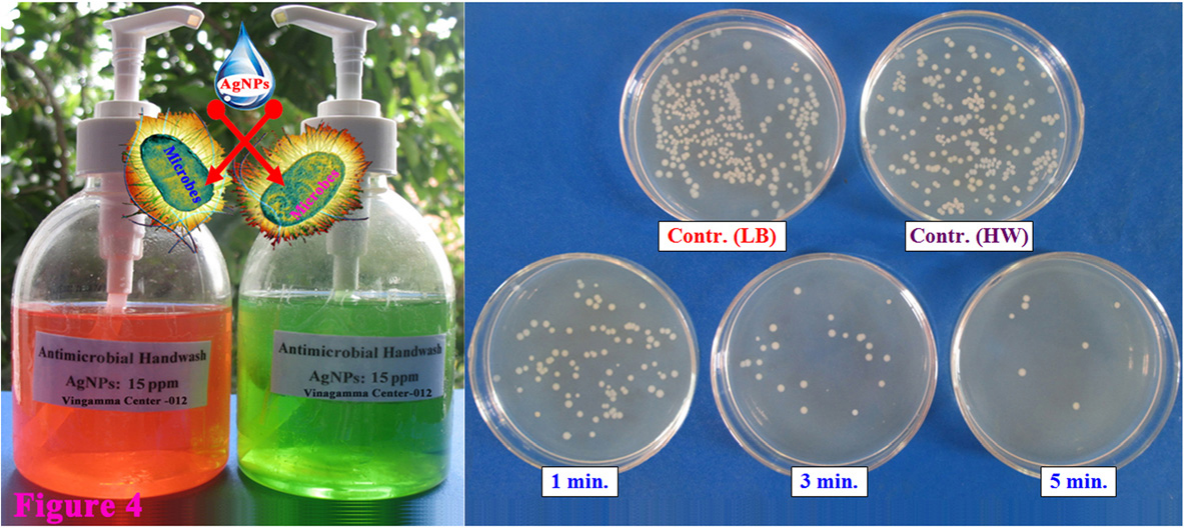
**Photograph of handwash containing AgNPs and the growth of *E. coli *** **in LB agar with time.**

Photographs of handwash solutions and bactericidal activity were showed in Figure 4. The handwash without AgNPs (HW) was almost non-antibacterial against *E. coli*; the *η* value reached approximately 6.2% only. The bactericidal efficiency with only 3-mg/L AgNPs diluted from the handwash solution against *E. coli* with a bioburden of approximately 10^7^ CFU/100 ml (*E. coli* infection is much higher in comparison with real conditions) was 74.6%, 89.8%, and 99.0% for 1, 3, and 5 min of contacting time, respectively (Table 2).

**Table 2:** **The bactericidal efficiency (*η *) of handwash/AgNPs with contacting time**

**Time**	***E. coli *** **(CFU/mL)**	***η *** **(%)**
Control (LB)	33.9 × 10^5^	-
Control (HW)	31.8 × 10^5^	6.2
1 min	86.0 × 10^4^	74.6
3 min	34.6 × 10^4^	89.8
5 min	3.3 × 10^4^	99.0

Wei et al. also reported the high bactericidal effect of AgNPs with sizes of 6 to 8 nm against *E. coli*, particularly the *η* value of 10-mg/L AgNPs which was approximately 99.9% for 2 min of contacting time [11]. Our obtained results revealed the high bactericidal activity of the as-prepared handwash that can be recommended to communities to apply for daily sanitary handwash for prevention of infectious diseases, such as diarrhea and enterovirus infection. Furthermore, Petica et al. reported that using Na-LS as co-stabilizer was highly effective for obtaining stable colloidal AgNP solution with very good antimicrobial and antifungal properties [26]. Concerning the environmental impact of AgNPs, it is also worth to note that the AgNPs in wastewater is almost completely transformed into Ag_2_S that has extremely low solubility and exhibits a much lower toxicity than other forms of silver [27,28]. Therefore, the as-prepared handwash/AgNP solution is expected to be stable for a longer duration and to maintain a bactericidal activity due to the presence of Na-LS as co-stabilizer. In addition, AgNPs eliminated from the handwash after use into wastewater will be transformed into Ag_2_S that is considered to have no significant impact to the environment [27].

## Conclusions

The colloidal AgNP solutions stabilized by PVA, PVP, sericin, and alginate were successfully synthesized by gamma Co-60 irradiation method. Results on antibacterial activity test demonstrated that AgNPs/alginate with an average size of 7.6 nm exhibited the highest antibacterial activity among the as-synthesized AgNP solutions. The as-prepared handwash with 15-mg/L AgNPs/alginate showed a high antibacterial efficiency with rather short contacting time. Thus, handwash/AgNPs can be potentially used as a daily sanitary handwash to prevent transmission of infectious diseases.

## Competing interests

The authors declare that they have no competing interests.

## Authors’ contributions

NQH came up with the idea. DVP and LAQ designed and set up the experimental procedure. NND, LQL, and BDD planed the experiments and agreed to the publication of the paper. NTKL conducted the size measurement of the as-prepared silver nanoparticles by TEM. NND, LQL, and LAQ performed the UV-vis measurement of the AgNP solutions stabilized by different polymers and evaluated the antibacterial efficiency of AgNP solutions and handwash solution containing AgNPs. NQH, LQL, and DVP analyzed the data, drafted the manuscript, revised the manuscript critically, and made a few changes. All authors read and approved the final manuscript.
